# The State-of-the-Art and Perspectives of Laser Ablation for Tumor Treatment

**DOI:** 10.34133/cbsystems.0062

**Published:** 2024-01-05

**Authors:** Yingwei Fan, Liancheng Xu, Shuai Liu, Jinhua Li, Jialu Xia, Xingping Qin, Yafeng Li, Tianxin Gao, Xiaoying Tang

**Affiliations:** ^1^ School of Medical Technology, Beijing Institute of Technology, Beijing 100081, China.; ^2^ School of Materials Science and Engineering, Hefei University of Technology, Hefei 230009, China.; ^3^ John B. Little Center for Radiation Sciences, Harvard TH Chan School of Public Health, Boston, MA 02115, USA.; ^4^ China Electronics Harvest Technology Co. Ltd., China.

## Abstract

Tumors significantly impact individuals’ physical well-being and quality of life. With the ongoing advancements in optical technology, information technology, robotic technology, etc., laser technology is being increasingly utilized in the field of tumor treatment, and laser ablation (LA) of tumors remains a prominent area of research interest. This paper presents an overview of the recent progress in tumor LA therapy, with a focus on the mechanisms and biological effects of LA, commonly used ablation lasers, image-guided LA, and robotic-assisted LA. Further insights and future prospects are discussed in relation to these aspects, and the paper proposed potential future directions for the development of tumor LA techniques.

## Introduction

Cancer is a serious health condition that has a negative impact on individuals, and it currently stands as the second leading cause of death globally. Statistical projections suggest that by 2040, there will be approximately 28.4 million new instances of cancer worldwide [1]. Tumor treatment mainly includes conventional surgical treatment, radiotherapy, chemotherapy, targeted therapy, immunotherapy, and optical therapy. These methods have exhibited rapid advancement in recent years. Optical technology has gained substantial traction within the fields of biology and medicine, offering advantages such as speed, real time, great controllability, and operability [2]. When it comes to medical applications [3], conventional therapies, including laser ablation (LA), photodynamic therapy (PDT), photothermal therapy (PTT), and photocoagulation therapy, have been integrated with laser techniques for clinical treatments.

The evolution of laser technology in the medical industry has encouraged its extensive utilization for cancer treatment [4]. Presently, lasers are crucial for the early diagnosis and treatment of cancers. The application of LA in place of surgical resection for treating tumors is becoming more widespread [5]. Laser therapy employs a focused narrow beam of intense light to eliminate or destroy cancer cells and abnormal cells with the potential to become cancerous. This approach aims to reduce or eliminate tumors or precancerous lesions. Selective laser wavelengths can be employed to precisely target tumor cells, exploiting their different wavelengths (or colors) compared to normal cells. Laser therapy is a localized method of treatment [6] that can be applied to specific body regions, and is frequently used to treat superficial cancers on the organ surfaces or inner linings of internal organs, which includes various types of cancer such as skin cancer [7], liver cancer [8], thyroid cancer [9], breast cancer [10], prostate cancer [11], and other types of cancer.

The integration of image-guided LA [12] and robot-assisted technology [13] has emerged as a current focus in both research and clinical applications. LA treatment has made diverse imaging-guided techniques, including endoscopic guidance, optical coherence tomography (OCT) guidance, magnetic resonance imaging (MRI) guidance, ultrasound (US) image guidance, and multimodal image [photoacoustic (PA), MRI-US] guidance. These image-guided treatment technologies hold immense potential across various tumor treatment domains. OCT offers high-resolution imaging data (on a micrometer scale) for visualizing and detecting superficial or early-stage cancers [14–16], thus serving as an invaluable real-time imaging tool and diagnostic technique. Effective recognition and precise measurement, facilitated by medical imaging, enable the accurate definition and delineation of target tissue [17], comparable to the “eye” of accurate identification, and empower LA to achieve enhanced precision and efficiency in therapeutic outcomes. Furthermore, the ongoing advancement in robotic technology has facilitated targeted and precise tumor treatment through robot-assisted laser therapy. Combined with exquisite end effectors carrying laser fibers, a new type of “hand” is installed on the robot in this way, using fibers to deliver energy to the targeted tumor tissue for therapeutic interventions.

The path of intelligent theranostics holds immense significance in the future medical development [17]. Research into intelligent analysis-guided LA therapy based on medical imaging and robot-assisted LA therapy grounded in reinforcement learning has gained substantial interest in recent years, in line with the continuous evolution of artificial intelligence and allied technologies. Additionally, equipping intelligent end effectors to guide optical fiber LA can generate noninvasive or minimally invasive therapeutic effects for intracavitary and natural cavity therapy. Employing flexible robot [18] to guide optical fibers in delivering light energy to target tissues for ablation treatment enables minimally invasive theranostics. Furthermore, the fusion of preoperative and intraoperative images or data is used to construct a three-dimensional (3D) model of the target tumor tissues for localization. Through surgical navigation technology [19] to guide intelligent treatment, an intelligent image-guided theranostic LA technology is established. Therefore, the integration of intelligent recognition, diagnosis, and treatment offers a potent solution for tumor LA treatment.

**Fig. 1.**
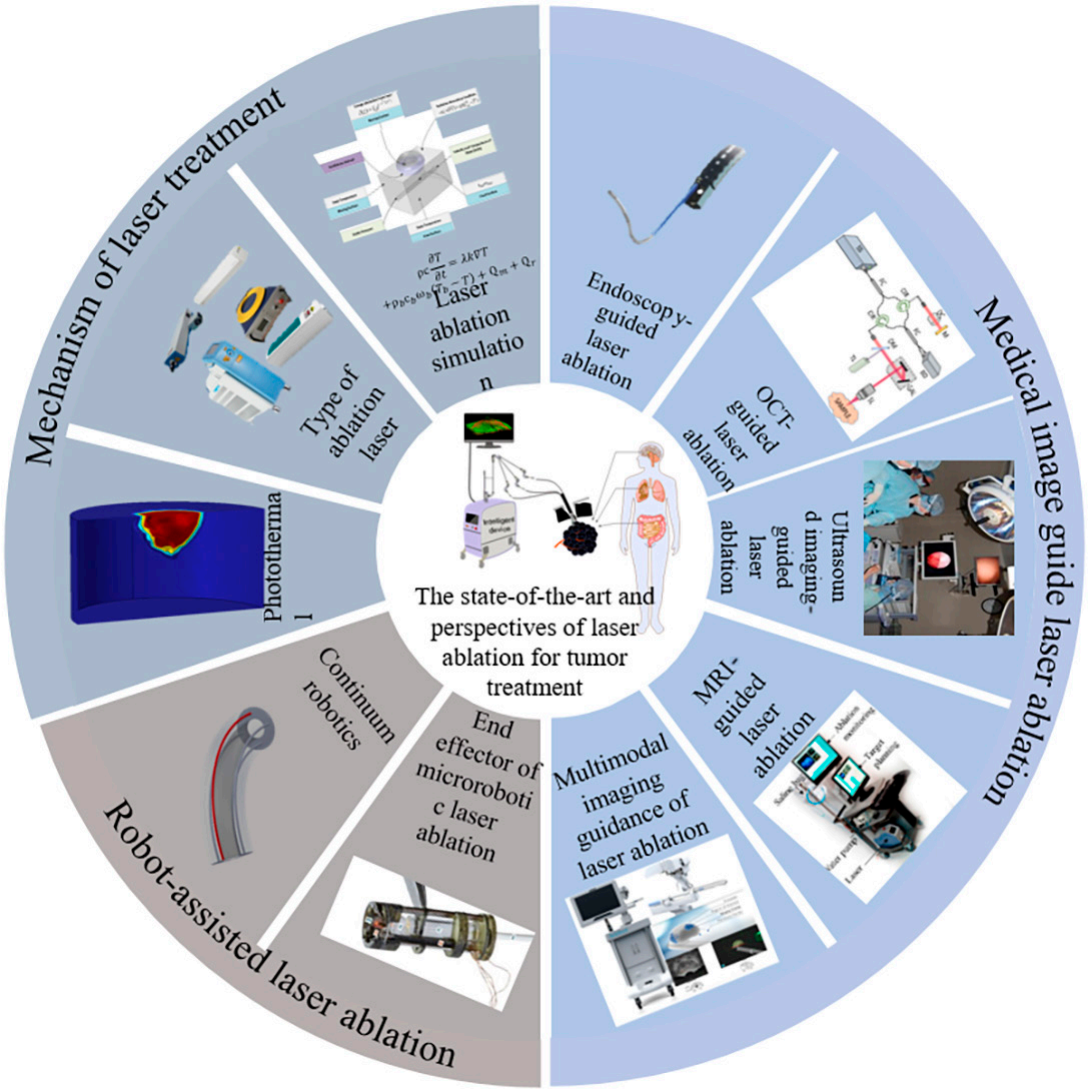
Research fields and hot frontiers of LA of tumor.

Intelligent multimodal image-guided LA therapy represents a key technical solution and path of development for tumor therapy research and clinical applications. In the course of tumor LA therapy, it is urgently necessary to find efficient strategies that synergize diagnostic and treatment modalities, multimodal information fusion techniques, and intelligent manipulation methods. This paper reviews the mechanism of LA therapy, recent advancements in image-guided LA, and prominent aspects of robot-assisted laser therapy and explores the future development direction of LA technology (Fig. 1). The contents included in this review are chiefly described in Table 1.

**Table 1.** Summary of LA for tumor treatment

Project	Research directions	Key features	Author/reference
Mechanism of LA therapy	Mechanism and biological effects of LA	Damaging tissues with light-generated heat	Walsh [20]
Dependent factors: laser wavelength, laser power, tissue optics, etc.		Müller and Roggan [21]
Tissue changes: damage, coagulation, carbonization, or ablation		Niemz [22]
Common laser types	CO_2_ laserNd:YAG laserDiode laserHo:YAG laserTm:YAG laser	Omi and Numano [7]Xu et al. [8]Mao et al. [29]Rashid et al. [40]Lan et al. [47]
Simulation of LA model	Pennes biothermal equationDamage equation, Arrhenius equationTransient numerical simulation model predicts LA behavior.Reconstruction of tissue temperature distribution based on Bayesian framework.	Pennes [55]Henriques [59]Mohammadi et al. [65]Schulmann et al. [66]
Image-guided laser ablation therapy	Endoscopic imaging, medical imaging (OCT, PA, etc.), US, MRI, and multimodal imaging can guide surgery by monitoring tissue damage during ablation in real time.		
Endoscopic-guided LA	Single-fiber bundle for endoscopic imaging and laser irradiation modulation.Dual-mode GNR near-infrared fluorescence imaging and ablationControlling tumor temperature with a thermal imaging sensor, visible light endoscope, and laser fiber.	Miyoshi et al. [12]Gournaris et al. [84]Ohara et al. [85]
OCT-guided LA	OCT-based real-time LA monitoring and control using double-clad fiberFibers for Tm and OCT beams, dual time reflectors for guide and therapy light.Integrated ablation and OCT catheter.Real-time LA guidance and monitoring employing independent fibers for laser and OCT beams.	Maltais et al. [92]Katta et al. [93]Kang et al. [94]Fan et al. [95]
US-guided LA	US-guided LA has been used in the clinical treatment of thyroid cancer, breast cancer, and pancreatic cancer, proving its feasibility.US-guided laser discectomy improves accuracy and safety.New three-fiber optical US probe.	Yong-Ping et al. [9]Perretta et al. [100]Di Matteo et al. [102]Hu et al. [105]Zhang et al. [106]
MRI-guided LA	MRI-guided LA has been used in the clinical treatment of epilepsy, osteoid osteoma, and prostate cancer, demonstrating its feasibility.LA temperature management using the preset temperature time curve to alter laser output power.New intraoral laser software robot MRI-guided minimally invasive techniqueAn MRI-compatible electromechanical needle navigation system.	Curry et al. [112]Barqawi et al. [114]Mehralivand et al. [115]Desclides et al. [120]Fang et al. [121]Knull et al. [122]
Multimodal image fusion-guided LA	A thermal diffusion-based tissue heat distribution model and real-time photoacoustic monitoring of the treatment region enable quick three-dimensional temperature mapping.A phased-array endoscope-based LA system combining ultrasound and photoacoustic imaging can simultaneously ablate tissue, identify tissue features, and monitor tissue temperature.MRI-US fusion has been recommended as the third imaging modality for real-time monitoring during prostate cancer treatment interventions.FDA has provided MRI-US fusion equipment through 510 (k)-approved targeted prostate biopsy.	Landa et al. [123]Basij et al. [124]Wimper et al. [125]
Robot-assisted laser ablation	Advanced LA surgical robot systems require the research of new robot catheters and precision micro-robot end effectors to reposition imaging sensors and laser actuators closer to the surgical area for stable and precise positioning and real-time monitoring of the ablation process.		
Micro-robot end effector	A laser scanner driven by magnetic force.A novel robot system with cable driven parallel mechanism.Oral robot surgery uses a piezoelectric beam-driven microelectromechanical integrated structure-based millimeter-level tip/tilt laser scanning system.This device is further miniaturized and can control the position and speed of fiber optic laser transmissionAn endoscope tip with stereo vision, illumination, and fixed-focus laser	Acemoglu et al. [133]Zhao et al. [134]Bothner et al. [135]York et al. [52]Kundrat et al. [53]
Continuum robotics (CR)	Tendon-driven CRMagnetic navigation CRSoft material-driven CR:Shape memory effect CRConcentric tubesConducting polymer-driven CRHydraulic pressure-driven CRHybrid actuation CRNovel biocompatible conductive polymer for visceral medical robotics	Kouh Soltani et al. [137]Liu et al. [139]Namazu et al. [141]Liu et al. [142]Piskarev et al. [143]Gopesh et al. [144]Zhang et al. [145]Chikhaoui et al. [80]

## Mechanism of Laser Treatment

LA involves the use of laser energy and a medium that transmits laser light within the tissue. The laser acts as a tool to convert light energy into heat within tissues. The interaction between the laser and biological tissues is influenced by the laser’s characteristics and its wavelength. The medium is typically a flexible optical fiber with a small diameter (0.2 to 0.8 mm) used for laser transmission in deep organs. By delivering laser energy to the target area, the tissue absorbs and converts it into thermal energy, resulting in the ablation of tumors.

### Basic principles and biological effects of LA

LA utilizes thermal energy generated by light to cause tissue damage [20]. Only the energy absorbed by the tissue can produce biological effects as light can propagate within tissue through reflection, transmission, scattering, or absorption. The tissue's ability to absorb light determines its heating capacity. Different tissue components and chromophores exhibit varying absorption characteristics based on wavelength, leading to specific interactions between laser light and tissues. The nature of the laser–tissue interaction is determined by a number of elements, including laser wavelength, laser power, exposure time, pulse duration, and repetition frequency (when utilizing pulse emission), optical properties of the target tissue [21].

The heat effect is the most important biological effect in the interaction between laser and tissue during LA. The amount of tissue burned at a particular wavelength is determined by the laser flux and irradiance. According to Niemz’s [22] research, at a typical body temperature of 37 °C, under a particular heating intensity, biological tissue cells can experience damage, coagulation, carbonization, or ablation depending on the degree and duration of tissue heating:

a. Heat therapy involves a modest temperature increase that elevates the tissue temperature to 41 to 44 °C within a few minutes, causing cellular damage, disintegration of cell membranes, and enzymatic denaturation.
b. Coagulation refers to a temperature range of 50 to 100 °C that the tissue reaches for about 1 s. The tissue necrosis occurs within this temperature range. Coagulation induces dehydration, leading to denaturation of protein and collagen, resulting in tissue contraction.
c. Ablation refers to the process where tissue reaches a temperature range of 100 to 1,000 °C in a short period of time (approximately ^1^/_10_th of a second), resulting in material loss and the removal of various components in the form of steam.

**Fig. 2.**
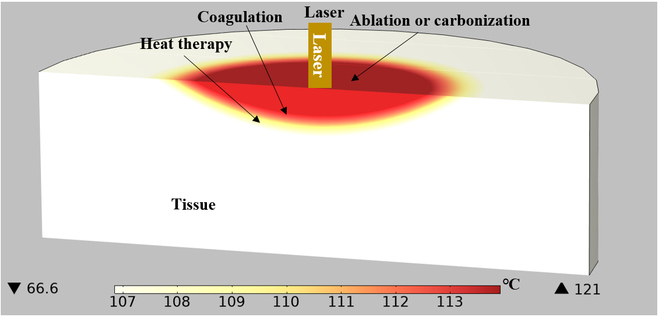
Distribution map of heat-affected area using laser radiation.

The various zones affected by heat resulting from the interaction between the laser and biological tissue are illustrated in Fig. 2.

### Ablation laser types

The effect of laser on tissues depends on the laser’s operational mode and wavelength. The most essential factor is the penetration depth, which is defined as the tissue depth necessary to absorb 63% of the input light intensity. Different laser types exhibit varying penetration depths, based on the laser’s wavelength and the characteristics of both the tumor and surrounding healthy tissue [23]. Many different lasers are utilized for tumor ablation to achieve diverse therapeutic outcomes. As a result, in practical practice, light wavelengths with substantial penetration depth are necessary to treat deep-seated cancers. During the surgical procedure, the operator must select the most suitable laser and its settings to achieve the therapeutic objectives [4]. Within the wavelength range of 940 to 1,100 nm, LA is suitable due to a favorable equilibrium between absorption and tissue penetration in this optical window, resulting in a broad solidification area.

The most common laser sources in optical therapy are shown in Table 2, which can shrink or destroy tumors and can be used in conjunction with endoscopes. CO_2_ laser can ablate the surface of the skin without penetrating deeper layers, so it can be used to remove superficial cancers, such as skin cancer [7,24]. In contrast, Nd:YAG laser has been the most widely used laser for decades due to its excellent penetration capability within deep tissues, which is used to treat liver cancers [8,25], breast cancers [10,26], osteoid osteoma [27,28], and others. Diode lasers offer wavelengths between 800 and 980 nm, and their tissue penetration is similar to that of Nd:YAG lasers. Presently, they have been used for treating prostate cancers [29–31], brain tumors [32–34], laryngeal cancers [35,36], intraocular cancers [37,38], traumatic fibroma [39], and so on. Ho:YAG laser has an average between output power of approximately 20 and 150 W and are commonly used for laser lithotripsy [40,41], prostate ablation surgery [11,42,43], orthopedic surgery [44,45], hysteroplasty [46], and so on. The average output power of Tm:YAG laser falls between approximately 20 and 100 W, and is commonly used for treating prostate hyperplasia [47–49], bladder cancers [50,51], and urolithiasis [52,53].

**Table 2.** Commonly used tumor ablation lasers and related parameters

Laser	Wavelength (nm)	Absorption chromophore	Target organ or tissue
CO_2_ laser	10,600	Water	Skin
Nd:YAG laser	1,064	Pigments, proteins	Liver tumor, breast cancer, osteoid osteoma
Diode laser	800–970	Pigments, water (range)	Prostate tumor, brain tumor, laryngeal tumor, intraocular tumor, traumatic fibroma
Ho:YAG laser	2,100	Water	Laser lithotripsy, prostate ablation surgery, orthopedic surgery, hysteroplasty
Tm:YAG laser	2,020	Water	Prostate hyperplasia, bladder tumors, and urolithiasis

Laser radiation can be achieved utilizing either continuous wave (CW) or pulse wave (PW) during the LA process. The laser power range in CWs is between 2 and 30 W, and the treatment duration varies from a few minutes to 20 min. In PW mode, laser energy is released periodically as a sequence of pulses, and the pickup power (i.e., the power level of each pulse) is higher than that of CW emitting lasers, but the average power remains equivalent. The suitable laser mode is determined by the specific treatment requirements and goals. CW mode is appropriate for long-term continuous treatment, whereas PW mode is suitable for circumstances requiring higher peak power.

### Simulation of LA

The modeling of physical processes in biological tissues irradiated by laser beam can be divided into three steps: modeling of laser energy deposition, temperature distribution, and damage in laser irradiated tissues [54]. Many heat transfer models have been developed to characterize the temperature distribution in laser-irradiated tissues. One of the most well-known models for estimating heat transmission in biological tissues is Pennes biothermal equation [55], which represents the temperature distribution within biological tissues. For constant thermophysical parameters, it has the following forms:

$$\begin{array}{c} \mathrm{\rho c}\frac{\partial T}{\partial t}=\lambda k\nabla T+\rho_{b}c_{b}\omega_{b}\left(T_{b}-T\right)+Q_{m}+Q_{r} \end{array}$$ (1)

*ρ* is the tissue density (kg cm^−3^), *c* is the tissue specific heat (J kg^−1^ °C^−1^), *k* is the tissue thermal conductivity (W m^−1^ °C^−1^), *T* is the tissue temperature (°C), *t* is the time (s), *ρ_b_* is the blood density (kg cm^−3^), c*_b_* is the blood specific heat (J kg^−1^ °C^−1^), ω*_b_* is the unit volume blood perfusion rate (kg m^−3^ s^−1^), *T_b_* is the arterial blood temperature at 37 °C, *Q*_r_ is the internal heat source term caused by photon absorption (W m^−3^), and *Q*_m_ is the metabolic heat production per unit volume, which can be ignored [56,57].

**Fig. 3.**
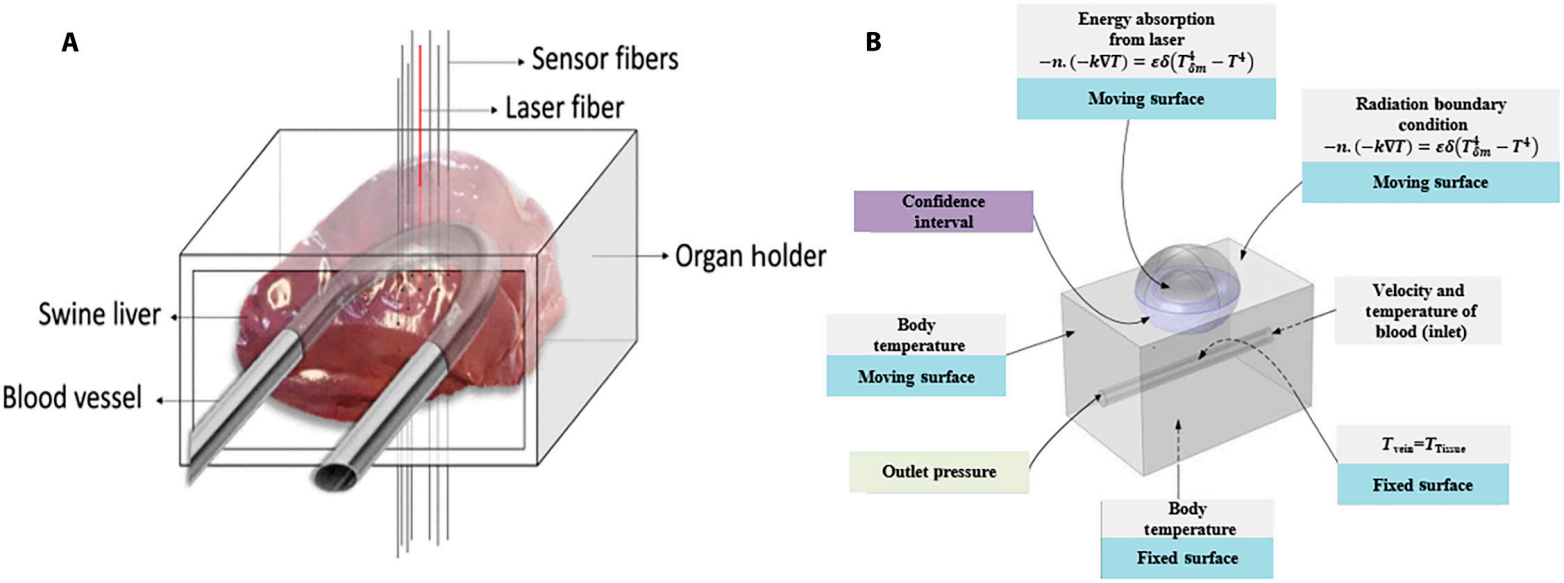
(A) Schematic diagram of organ scaffold for pig liver in LA. Reproduced with permission from [58]. (B) Schematic diagram of the geometric shape of healthy tissues, including tumors, blood vessels, and their boundary conditions. Reproduced with permission from [65].

Vita et al. [58] employed fiber optic sensors to measure temperature inside the livers of perfused pigs undergoing LA. As illustrated in Fig. 3A, artificial blood arteries are employed to replicate blood perfusion in the isolated liver. Comparisons were made between temperature distribution in two different spatial configurations of blood vessels and fibers to study the effect of blood perfusion on LA.

The final step in analyzing the tissue heating process is to estimate the extent of damage, typically achieved using the Arrhenius equation [59–61], which can obtain the degree of tissue damage during the treatment process, expressed as follows:

$$\frac{\mathrm{d\alpha}}{\mathrm{dt}}=\text{Aexp}\left(-\frac{∆E}{RT}\right)$$ (2)

*A* is the frequency factor (s^−1^) and ∆*E* is the activation energy of irreversible damage reactions (J mol^−1^). These two parameters depend on the type of organization. *R* is the general gas constant. The value is 8.31 J mol^−1^ K^−1^, and *T* is the absolute temperature. The expression Θ*_d_* for the proportion of necrotic tissue is as follows:

$$\Theta_{d}=1-\text{exp}\left(\;-\alpha \;\right)$$ (3)

The main numerical simulation methods for LA include finite difference, finite volume, boundary element, spectral analysis, numerical integration transformation, and others. There are several main factors that affect LA results, including laser wavelength, power, and treatment duration [62]; mechanical, thermal, and optical properties of the tissue [63]; the presence and quantity of blood flow [64]; and the optical emission characteristics of the applicator [21].

Mohammadi et al. [65] created a transient numerical model to predict the behavior of LA under various settings, and developed a thermal mechanical model, as illustrated in Fig. 3B. Experimental data from pig liver tissue were employed to assess its applicability and efficacy, revealing that laser power and exposure time are the most critical variables impacting ablation phenomena. However, it is the result of certain geometric forms and situations; thus, individual patient scenarios should be considered in optimizing therapeutic application.

Schulmann et al. [66] proposed a fast data assimilation Bayesian framework for real-time reconstruction of the spatiotemporal distribution of tissue temperature under laser irradiation. They used unscented Kalman filters to associate the predictions of the heat transfer model with sparse real-time temperature measurements obtained from fiber-optic thermometers. This approach further optimizes the 3D degree of the structural domain while considering tissue heterogeneity and physiological heat sources, facilitating automatic LA control in the clinical setting. During the LA process, Korganbayev et al. [67] achieved constant temperature control through a closed-loop feedback proportional integral differential controller. This controller adjusts the laser power based on real-time measurement, thereby maintaining the tissue temperature within a constant target range. These studies aim to enhance the control and monitoring of LA processes. By real-time reconstruction of tissue temperature distribution and achieving automatic control, medical professionals can more accurately adjust laser parameters to achieve the desired therapeutic effect. These methods provide important support for the accuracy and safety of LA, further advancing the application of this technology in the clinical practice.

## Medical Image Guide LA

LA is a minimally invasive technique that destroys tumors within solid organs by directing low-power laser energy into the tissue. As an alternative method to surgical resection, LA is increasingly gaining recognition in the field of tumor treatment. Although being associated with certain complications, LA stands as the only accessible treatment option for patients who cannot tolerate general anesthesia, as it is a minimally invasive or noninvasive method that can be performed under local anesthesia.

In the course of LA treatment, it is important to take into account all factors influencing the generated ablation effect while safeguarding the surrounding healthy tissues. Therefore, real-time assessment of tissue damage throughout the ablation process is critical for guiding surgery process, enhancing surgical efficiency, and defining the endpoint of laser energy deposition [68]. Histology has typically been regarded as the gold standard for distinguishing tumor types in clinical diagnosis. Histological analysis of biopsy, on the other hand, is an intrusive and time-consuming procedure that can result in misinterpretation due to sample variability. Consequently, noninvasive diagnostic procedures utilizing real-time imaging are preferred. Various approaches, such as endoscopic imaging, medical imaging (including OCT, photoacoustic tomography, etc.), US/endoscopic US (EUS), MRI, and multimodal imaging, can all be used to guide and monitor LA progress. Pacella and Mauri [69] summarized the technical characteristics and clinical application of image-guided LA, which has been used in treating lung cancers [70], thyroid cancers [71], brain cancers [72], and prostate cancers [73].

### Endoscopy-guided LA

The combination of endoscopic access and laser therapy has been successfully applied in a wide range of clinical applications [74]. Compared to open surgery and laparoscopic access, endoscopic access minimizes the need for incisions and sutures [75], resulting in less patient trauma. Furthermore, laser therapy, characterized by its noncontact nature, exceptional selectivity, and resolution, can be further automated through the utilization of robot technology [76] and numerical simulation [77–79]. Researchers have developed micro-endoscopic tips with enhanced flexibility and functionally [80–82] in order to expand the scope of endoscopic applications. These micro-endoscopic tips have a smaller outer diameter and greater mobility, allowing laser treatment in narrow and complex abdominal structures.

Mo et al. [83] designed a flexible endoscope system with 2- and 12-mm-diameter flexible manipulators, combined with an integrated camera. Endoscopes can provide visual feedback, while flexible manipulators can transmit and control laser beams through optical fibers. Flexible manipulators and endoscopic movements have been decoupled through the specialized mechanical structures and novel cable configurations. This provides an effective solution for laser-assisted endoscopic surgery within confined spaces. In subsequent research, further studies are required to evaluate the surgical applicability of in vitro and in vivo tissue ablation.

**Fig. 4.**
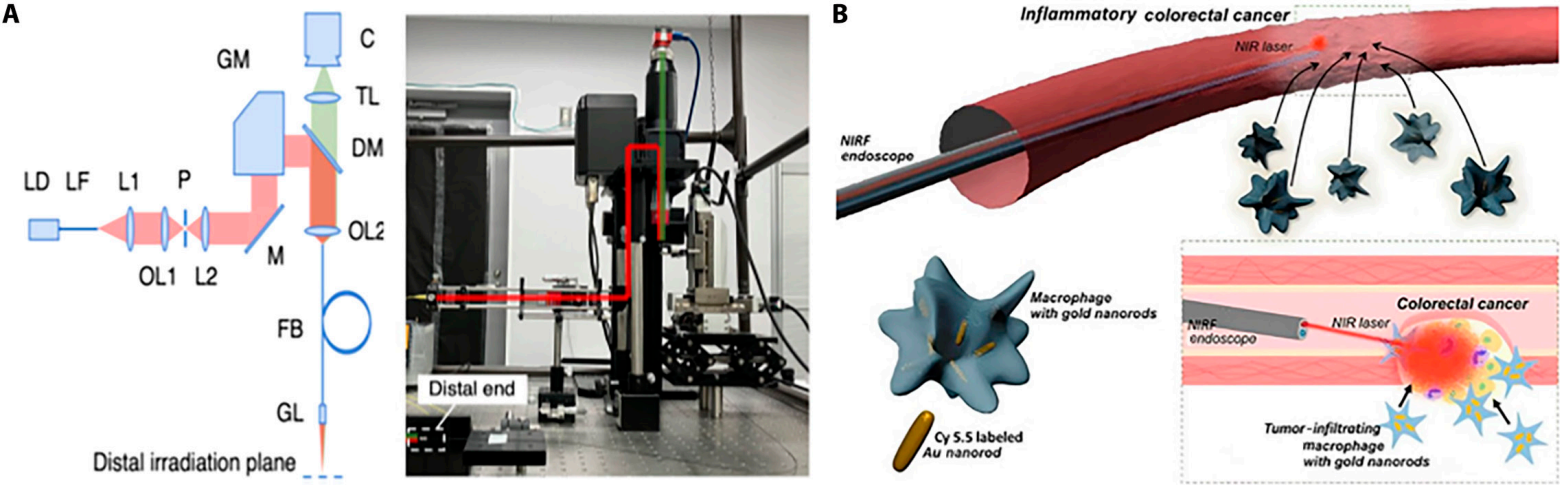
Endoscopy-guided LA diagram. (A) Schematic diagram of endoscopic image-guided laser treatment system based on fiber bundle laser steering. Reproduced with permission from [12]. (B) Schematic diagram of NIRF endoscopic image-guided PTA for bimodal GNR treatment of colorectal cancer. Reproduced with permission from [84].

Miyoshi et al. [12] created a novel endoscopic image-guided laser therapy system. As illustrated in Fig. 4A, this system uses a single-fiber bundle to capture endoscopic images while simultaneously modulating the laser irradiation region, allowing for minimally invasive endoscopic laser treatment in narrow anatomical sites. The approach achieves laser-guided steering by manipulating the position of magnetic core near the laser input, which has some limitations and might be improved. Additionally, this technology lacks real-time temperature monitoring during the ablation process, which constitutes a limitation.

As illustrated in Fig. 4B, Gournaris et al. [84] developed a near-infrared fluorescence (NIRF) endoscopic image-guided photothermal ablation (PTA) approach for the effective treatment of colorectal cancer (CRC). The bimodal gold nanorods (GNRs) were created by combining rod-like gold nanoparticles and Cy5.5 NIR fluorescent molecules, which can not only facilitate NIRF imaging but also exhibit an efficient photothermal effect upon near-infrared wavelength laser irradiation through an endoscope. Precise ablation of polyps was achieved utilizing NIRF endoscopic image-guided PTA and bimodal GNRs. However, this method also lacks real-time temperature monitoring during the ablation process.

Endoscopic LA has the advantages in terms of minimal invasiveness, shortened procedure duration, and enhanced precision [75]. However, there are limitations in the ablation process due to the current inability to achieve real-time temperature monitoring. Traditional treatment procedures often require temperature measurement using a thermal imager placed outside the body, thereby inhibiting temperature control and potentially resulting in damage to healthy tissues due to overheating. In response, Ohara et al. [85] created a thermal imaging endoscopic system to regulate tumor temperature for treating peritoneal dissemination. This system incorporates a thermal imaging sensor, a visible light endoscope, and a laser fiber. The researchers established a tumor temperature control system based on feedback control and a Gaussian function for estimating tumor temperature. This approach is noninvasive and has a good therapeutic efficacy. Experimental results indicate that the system maintains the essential stability of tumor temperature required for inducing tumor necrosis, resulting in a therapeutic impact. This work offers a new technique for temperature management in endoscopic laser therapy, which improves treatment accuracy and controllability. However, additional study and testing are required to verify the system’s effectiveness and safety.

While endoscope allows for precise localization of pathological areas, achieving real-time and accurate control of tissue temperature during LA therapy via endoscopic imaging remains a challenge. This is due to the need for external temperature sensors for monitoring, which poses a significant hurdle for miniaturizing endoscopes. Hence, one of the future directions is to develop compact, efficient, and precise temperature sensors, which would facilitate real-time and accurate control of tissue temperature during tumor LA.

### OCT-guided LA

OCT is a high-resolution imaging technology that is label free, real time, noninvasive, and radiation free. It offers the ability to provide real-time imaging at the micrometer level, which has clear benefits in guiding surgical ablation procedures. Because OCT imaging uses fiber optics, it is simple to connect with handheld surgical probes, laparoscopes, catheters, and endoscopes [86]. OCT imaging, unlike US imaging, is noncontact and can be imaged through air. Furthermore, OCT systems are often small and portable, making them simple to transport and operate. Finally, OCT has a swift imaging speed and can acquire image data on huge portions of tissue in a brief timeframe. OCT imaging has major application value in guiding the LA process because it is based on the optical backscattering characteristics of tissues and can detect the LA features of tissues. It can continuously track the impact of laser energy on tissues in real time and provide valuable guidance and feedback.

Yu et al. [87] investigated the use of OCT for real-time guidance in retinal surgery, showing its viability for real-time guidance. Several research teams have employed B-scan OCT feedback to track the tips of various instruments such as micropipettes [88], fixtures [89], and injection pipettes [90]. These studies indicate that OCT can be utilized to successfully guide various robot procedures in real time. Boppart et al. [91] performed extensive ablation studies on more than 60 components of five different types of isolated rat organ tissues. Their work discovered that OCT can swiftly identify tissues and guide ablation positions, as well as capture dynamic changes in tissue optical reflectance induced by laser heating before, during, and after laser irradiation. These findings highlight OCT’s potential as an image-guided tool in tumor ablation.

**Fig. 5.**
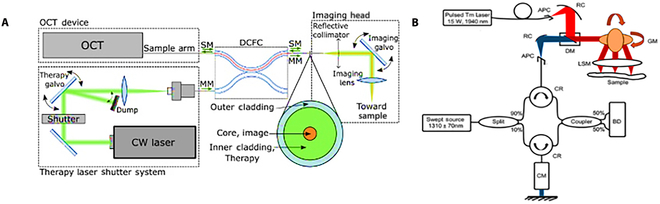
Schematic diagram of OCT-guided LA system. (A) Schematic diagram of endoscopic OCT-guided LA system. Reproduced with permission from [92]. (B) Schematic diagram of OCT and nanosecond-pulsed thallium TM laser tumor resection system. Reproduced with permission from [93].

As illustrated in Fig. 5A, Maltais et al. [92] created an endoscopic OCT-guided LA system capable of real-time monitoring and control of laser therapy. This system employs OCT technology to perform simultaneous imaging and treatment using a double-clad fiber. The system evaluates tissue coagulation depth using speckle intensity correlation and introduces noise reduction and motion correction methods to enhance monitoring precision and stability. It can detect the depth of the coagulation layer in real time and turn off the therapy laser automatically to obtain the desired treatment outcome. In the future, this technique opens the possibility for high spatial and temporal resolution, and single-fiber endoscopic-guided laser therapy.

Katta et al. [93] designed a tumor excision system that combines OCT and a nanosecond-pulsed thallium (TM) laser system. As shown in Fig. 5B, this system utilizes optical fibers to transmit TM and OCT beams, aligning them using reflection collimators and dual-time reflectors. Beam focusing is achieved through the galvanometer reflector in the focal plane behind the scanning lens. The simulation programs COMSOL and Zemax were utilized by the study team to build a model of this process. The simulated results for tissue removal rate closely aligned with experimental outcomes. The OCT-TM system emerges as a promising tool for cancer surgery. Another notable approach, by Kang et al. [94], introduces a temperature monitoring LA system based on OCT. Using a multifunctional integrated catheter for both LA and OCT imaging, this high-resolution and high-speed system enables real-time monitoring of the LA process. The design of this multipurpose integrated catheter allows LA and OCT imaging to be conducted in the same device, making real-time monitoring more convenient and efficient.

**Fig. 6.**
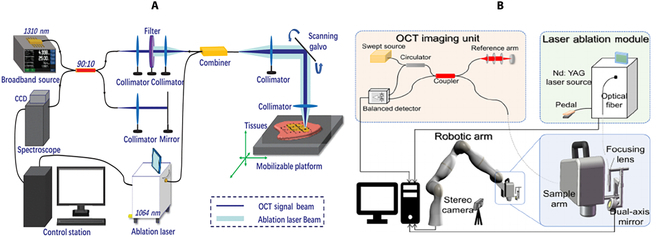
(A) Schematic diagram of scanning mode for common optical path. Reproduced with permission from [96]. (B) Intelligent optical theranostic system for tumor resection. Reproduced with permission from [97].

Recently, our team pioneered a new spectral domain OCT (SD-OCT) real-time guidance and monitoring LA system, aiming to achieve accurate treatment of soft tissue [95]. This system combines OCT and LA, using a separate optical path and manually aligning endoscopic images for operation. In this system, OCT initially generates tumor images, followed by real-time monitoring of the LA process. The system has been tested through phantom and in vitro pig brainstem experiments, demonstrating its efficiency and feasibility of accurate resection of lesions. In addition, we conducted further research and proposed a scanning mode with a common optical path to achieve OCT-guided LA [96], as illustrated in Fig. 6A. We also designed an intelligent optical theranostic system for tumor resection [97], as illustrated in Fig. 6B, incorporating automatic lesion localization and LA functions. Phantom and animal experiments were conducted for systematic verification. The robot can reach the planned position with high accuracy, approximately 1.16 mm. The accuracy of tissue classification using OCT images reaches 91.7%, and a minimal error of approximately 0.74 mm for OCT-guided automatic LA. This system offers real-time monitoring of automated operations with enhanced accuracy and efficiency compared to traditional cancer theranostics.

**Fig. 7.**
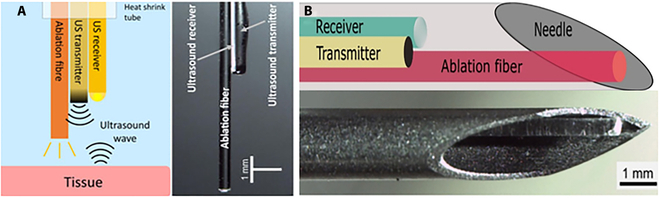
(A) Schematic diagram of OpUS imaging and LA system. Reproduced with permission from [106]. (B) Schematic of the fiber device. Reproduced with permission from [107].

OCT-guided LA surgery introduces improved controllability and precision in interactions between tools and tissues, facilitating precise surgical margins while minimizing collateral damage to neighboring structures such as blood vessels, nerve tissues, or other adjacent tissues. Experimental findings from various research teams have indeed demonstrated the potential of OCT-guided laser procedures in accurate tumor resection. Nonetheless, further research and validation, particularly in clinical settings, are imperative to assess its safety, feasibility, and efficacy. Despite these promising prospects, challenges persist in fully integrating OCT and LA within endoscopic systems. Future endeavors could explore co-optic pathways for OCT and therapeutic lasers, enabling their registration at the robotic end of endoscopes. The development of reliable control methodologies would be instrumental in achieving precise tissue ablation.

### US imaging-guided LA

US imaging can produce high-quality images that are simple to use, adaptable, radiation free, and affordable. It is extensively used to guide the ablation of parenchymal organs and tissues, particularly within the abdomen and pelvic cavities, due to its ability to provide real-time visualization of lesion size and its relationship with neighboring structures.

Lim et al. [98] investigated the acute reaction of pancreatic tissue following EUS-guided cylindrical interstitial laser ablation (CILA) in a pig model, aiming to compare the effects of different energy levels on pancreatic tissue ablation. The study found that EUS-guided CILA, facilitated by a diffusion applicator, produces consistent and predictable ablation under real-time EUS guidance. It demonstrated that adjusting the total energy delivered allows for control over the ablation area. The results show that EUS-guided CILA is a feasible method for effective pancreatic cancer treatment.

US/EUS-guided LA has made significant progress in clinical applications. Clinical application of this method is highlighted in Table 3 across various diseases, including thyroid cancers [9,99], breast cancers [100,101], and pancreatic cancers [102]. These studies indicate that during follow-up examinations, US/EUS-guided LA successfully achieved the treatment goal, with complete lesion ablation and no residual tumors observed. These findings validate the safety and feasibility of US/EUS-guided LA as a potential alternative for patients unsuitable for traditional surgical approaches [101]. However, longer-term follow-up studies and larger-scale research are indispensable to assess the long-term efficacy and safety of this method.

**Table 3.** US imaging-guided LA

Author	Tumor (number of patients)	Device	Guided image	Follow-up/complications
Yong-Ping et al. [9]	Thyroid cancer (43)	MT,EXL	US	23.47 ± 6.50 months, no complications, the ablation lesion disappeared
Zhang et al. [99]	Thyroid micro-cancer (12)	MT,EXL	US	19.75 ± 11.46 months, all ablated lesions completely disappeared or only scar strips remained
Perretta et al. [100]	Breast cancer (11)	MTM9,EXL	US	32.6 ± 9.2 days, no tumor residue (10), 1/11 residual cancer (1)
Nori et al. [101]	Breast cancer (12)	ML70,EXL	US	28.5 months, there was no evidence of local or distant recurrence in patients
Di Matteo et al. [102]	Pancreatic cancer (9)	EUE,EXL	EUS	Median survival period, 4.29 months

Intraoperative US is critical for real-time monitoring of LA. It can monitor the range and size of the echo produced by the gas in the ablation area, guiding complete ablation while minimizing thermal damage. However, traditional US imaging before and after LA therapy often exhibits low monitoring efficiency due to frequent deviations between the measured tumor size in the images and predictions [103]. Furthermore, traditional US has limitations in measuring the efficacy of ablation following surgery due to the interference of strong echo generated by the gas in the ablation zone. Contrast agents offer a solution by enhancing the backscattered echo in US imaging, hence boosting the resolution, sensitivity, and specificity of US diagnosing outcomes [104]. These agents can substantially increase contrast within the tumor area, allowing for a more accurate assessment of ablation effects. With the implementation of this technique, the monitoring of US imaging in LA treatment becomes more reliable and effective.

Hu et al. [105] offered US-guided percutaneous laser discectomy as a new approach for treating cervical root pain. Compared with traditional surgery, real-time imaging allows for enhanced accuracy and safety, which is beneficial in identifying the corresponding sections of the cervical intervertebral disc and preventing vascular injury. The goal of this study is to introduce a noninvasive treatment plan alternative to existing conventional surgical techniques. US-guided laser resection can result in more accurate and safe surgical treatments, as well as better therapeutic outcomes. This study is of significant importance in enhancing the quality of life of patients suffering from cervical root discomfort.

Figure 7A shows a novel type of optical ultrasound (OpUS) probe created by Zhang et al. [106]. This device can perform LA as well as real-time complete optical US imaging for ablation monitoring. The probe is made up of three optical fibers with a total equipment diameter of less than 1 mm. This probe features two fibers for ultrasonic transmission and reception, and one for LA light transmission. The authors performed ablation monitoring experiments on isolated porcine liver and heart tissue, and employed a segmentation algorithm to track ablation depth and identify lesion boundaries. The study results demonstrate the potential of the OpUS probe in real-time guided minimally invasive ablation procedures. Additionally, the integration of this probe into a medical needle (Fig. 7B) for performing laser interstitial thermal therapy (LITT) and monitoring on isolated lamb kidneys demonstrates its feasibility [107].

The novelty of this study lies in the combination of LA and complete optical US imaging, which allows for simultaneous operation of both functionalities within the same probe. This integrated design simplifies and expedites the surgical process, and the real-time imaging capability of the OpUS probe assists clinicians in properly locating the ablation area and lesion boundaries, improving surgical accuracy and safety. Further research and practice are warranted to explore the performance, feasibility, and applicability of this probe across various tissue types and lesion scenarios. This will contribute to the broader adoption of OpUS probes in minimally invasive ablation surgery.

In recent years, patch or wearable US imaging [108,109] has made significant progress in deep tissue imaging, with a remarkable resolution of 0.5 mm. These techniques yield clear results ever in deep tissues. Wearable US imaging offers the advantages of easy disassembly and reassembly. By adhering to the surface of biological tissues, it allows for prolonged observation of tissue changes [110]. The fusion of portable or patch-type US imaging-guided interventional fiber LA will provide huge technical support in the ablation treatment of deep-seated tumors.

### MRI-guided LA

In recent years, MRI has been widely developed and used as a guiding tool. It boasts soft tissue contrast, spatial and temporal resolution, and imaging capabilities that are practically real time. MRI exhibits high sensitivity to temperature changes within tissues, making it excellent for monitoring the thermal effects of laser energy on tissues compared to traditional interventional monitoring techniques such as computed tomography (CT) and US. MRI can scan multiple planes and possesses a vascular emptiness effect and an inflow-related enhancement effect without the need of contrast agents or radiation. It can position piercing guide needles and optical fibers in three dimensions. MRI is useful in tumor detection as it enables tumor localization and volumetric assessment and provides accurate guidance throughout the biopsy process. Furthermore, during minimally invasive treatment, MRI can provide real-time tracking of treatment effectiveness. The MRI-based temperature measurement system offers outstanding spatial resolution and accuracy, making it a promising technology approach. However, several restrictions, including the high cost of MRI equipment, complex operation [4], and stringent patient cooperation requirements.

LA technology compatible with MRI employs optical fibers to deliver laser energy through a sapphire tip to the core of brain lesions. The laser induces concentrated thermal damage by heating the surrounding tissues. Real-time MRI temperature measurement is utilized to monitor and regulate the ablation process to ensure accuracy and safety. This system captures real-time temperature data, enabling physicians to monitor temperature changes in the treatment area and adjust the ablation accordingly [111].

In Table 4, some clinical applications of MRI-guided LA are reported, and this technology has been applied in monitoring the ablation treatment of epilepsy [112] and osteoid osteoma [28]. Especially in the surgical treatment of drug-resistant epilepsy in children [113], MRI-guided LITT has demonstrated the potential of being a minimally invasive alternative therapy.

**Table 4.** MRI-guided LA

Author	Tumor (number of patients)	Device	Follow-up/complications
Curry et al. [112]	Hamartoma (71)	NeuroBlate system	23% need several ablations
Arocho-Quinones et al. [113]	Pediatric brain tumors (86)	Visualase system (Medtronic) [156], NeuroBlate system (Monteris Medical) [157]	80.6% of patients had stereotactic LA to reduce tumor volume after 24 months
Seemann et al. [28]	Osteoid osteoma (31)	Philips Panorama HFO 1.0T MRI system	Short-term follow-up (97% patient satisfaction) Long-term follow-up (100% pleased)
Barqawi et al. [114]	Prostate cancer (7)	GE Signa HDxt 3.0 T scanner	Six patients did not experience any surgical complications during the 1-year follow-up period
Mehralivand et al. [115]	Prostate cancer (15)	Philips Achieva 5.1T scanner	Seven patients experienced recurrenceTwo patients received FLA salvage treatment at 3 and 20 months, respectively

MT, MyLab Twice Ultrasound system; MTM9, MyLab 9 XP Ultrasound system; ML70, MyLab 70 XVG;

EUE, EG-3830UTK Ultrasound endoscope; EXL, Echolaser X4 laser (Nd:YAG laser).

In a phase I prospective cohort study, MRI-guided focused laser ablation (FLA) has been shown to be a feasible and safe option for treating patients with localized low-to-medium risk prostate cancer [114–117]. Recent study investigated the possibility of FLA for recurrent prostate cancer. MRI-guided FLA provides exceptional soft tissue contrast and anatomical visualization to facilitate treatment planning and targeting. Therefore, MRI-guided FLA offers significant imaging advantages over other surgical and ablation techniques that rely on transrectal US guidance and monitoring for treatment [118]. In addition, a study has confirmed that MRI-guided FLA can serve as a feasible salvage treatment for recurrent high-intensity focused ultrasound (HIFU) guided by MRI [119].

Desclides et al. [120] have developed a real-time automatic temperature control method for LITT, which utilizes MRI to measure tissue temperature and adjusts laser output power based on a predetermined temperature time curve. This procedure was implemented on a clinical MRI scanner and assessed using gel and animal models. Results indicate that this approach can precisely and reliably regulate tissue temperature, which should enhance the clinical efficacy and safety of LITT treatment.

**Fig. 8.**
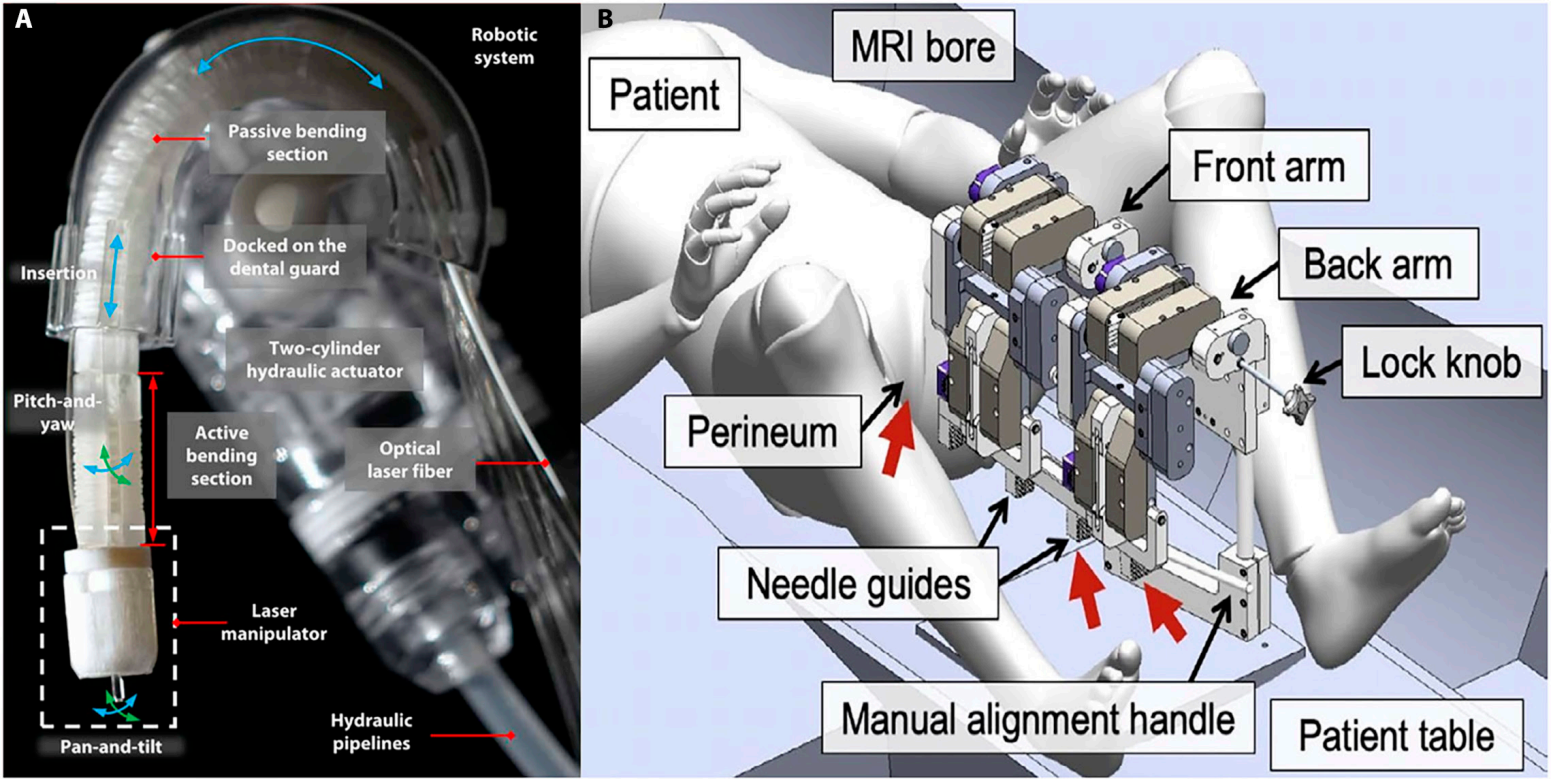
(A) Schematic diagram of a five-degree-of-freedom endoscope. Reproduced with permission from [121]. (B) Computer-aided design isometric model of MRI-compatible electromechanical needle guidance system for prostate focal LA needle intervention. Reproduced with permission from [122].

As shown in Fig. 8A, Fang et al. [121] have devised a new type of software-driven robot for intraoral laser minimally invasive procedures guided by MRI. Comprising both soft and rigid structures, this robot possesses five degrees of freedom and operates safely and flexibly within MRI-confined space. To accomplish precise laser positioning and control, the robot is propelled by a fluid of microvolume. Traditional intraoral surgery necessitates the use of specialized instruments, whereas laser minimally invasive procedure can achieve precise tissue cutting through the use of precisely positioned laser beams, thereby minimizing harm to the surrounding normal tissues. The future direction of development is to use lens tail fiber as the OCT sampling arm, coupling OCT with ablation settings, which can provide tissue depth images with micrometer resolution, thereby enhancing visibility and precision during surgical procedures and enabling physicians to better understand and treat oral lesions.

Knull et al. [122] developed an MRI-compatible electromechanical needle navigation system for thermal FLA of local prostate cancer. This system features a remotely driven four-degree-of-freedom transperineal puncture localization and needle guidance mechanism, along with an adaptable needle catheter, as shown in Fig. 8B. Using a tissue-simulated prostate model, this system demonstrated needle localization and delivery under mechatronic MRI guidance. This MRI-compatible electromechanical needle navigation system enhances surgical precision and safety by enabling more precise and controlled needle positioning during procedures.

### Multimodal imaging-guided LA

**Fig. 9.**
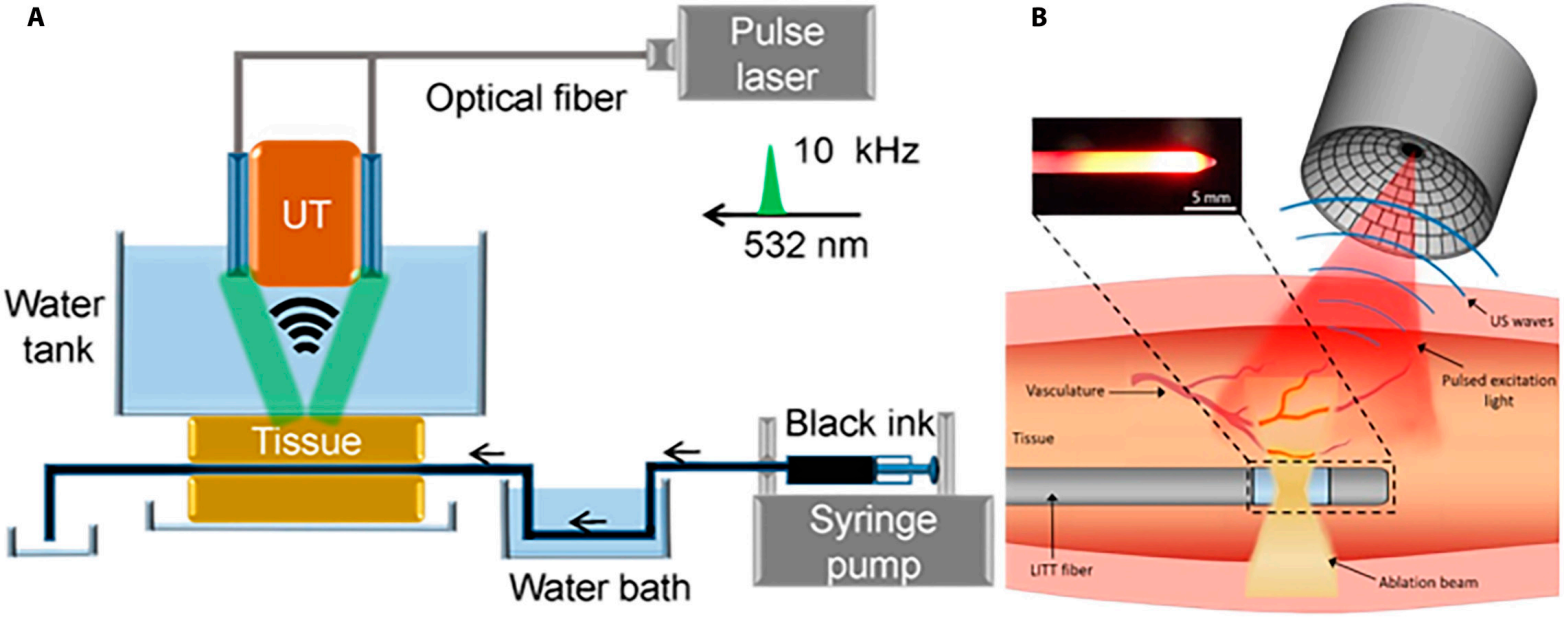
(A) PA imaging principal diagram. Reproduced with permission from [155]. (B) Layout of experimental settings. Reproduced with permission from [123].

PA imaging is a technique that utilizes pulsed laser stimulation to induce thermoelastic expansion and subsequent relaxation to generate sound waves. The underlying principle is shown in Fig. 9A. Due to its temperature sensitivity, PA imaging can be used to monitor heat treatment and draw temperature distribution within biological tissues. Landa et al. [123] proposed a rapid 3D temperature mapping method through real-time PA sensing of the treatment area, coupled with a tissue heat distribution model based on thermal diffusion. This method can map the development of temperature field during laser-induced thermotherapy, which helps to improve the safety and efficacy of optical therapy. The layout of their experimental setup is shown in Fig. 9B. Basij et al. [124] have developed an LA treatment device that integrates US and PA imaging guidance with traditional phased-array endoscopes. This device uses a phased-array endoscope for tissue ablation while simultaneously discerning tissue characteristics and monitoring tissue temperature. Applying this US/PA-guided theranostic system in clinical setting can greatly minimize complications. The combination of PA imaging and therapy provides the ability for simultaneous imaging and manipulation, leading to safer and more effective treatment. The application of this technology helps to improve the accuracy and outcome evaluation of treatment, and has broad potential in clinical practice.

With the advancement of technology, the MRI-US fusion system and its associated technology have undergone significant progress. MRI-US fusion has been recommended as the third imaging modality for real-time monitoring during prostate cancer treatment [125]. The advantage of this fusion technology lies in its potential to offer both medical professional and patients improved accessibility to services while potentially reducing costs. Among the available approaches, the simplest and possibly least accurate method is “cognitive fusion” technology. This technique involves utilizing multiparametric MRI (mpMRI) for pre-procedure visualization, followed by real-time US guidance during biopsy procedures. However, this method is susceptible to variations based on the operator’s expertise, and in situations involving small lesions, achieving accurate sampling might pose challenges [126]. Table 5 lists the devices approved by the U.S. Food and Drug Administration (FDA) in recent years to provide MRI-US fusion for targeted prostate biopsy through the 510 (k) pathway.

**Table 5.** FDA-approved MRI-US fusion devices through 510 (k) pathway

Manufacturer/trade name	US image acquisition	Biopsy path	Tracking mechanism	Year of FDA approval	Comments
Biobot Surgica/iSR'obot Mona Lisa 2.0	Robotic arm moving for US scanning	Transperineal	8-axis mechanical arm	2021	Integrated with existing US device, importing MRI images for registration
Eigen/Artemis	Manual US scanning	Transperineal/transrectal	Mechanical arm with encoders	2016	Integrated with existing US device, importing MRI images for registration
Invivo Corporation/Uronav (Version 2.0)	Manual US scanning	Transperineal/transrectal	Electromagnetic measurement system	2015	Integrated with existing US device, importing MRI images for registration
KOELIS/TRINITY	Specially designed US probes	Transperineal/transrectal	Organ-based tracking fusion	2016	Provide real-time 3D MRI-US fusion
Focal Healthcare/Fusion Bx 2.0	Manual US scanning	Transperineal/transrectal	Mechanical arm with encoders	2018	Integrated with existing US device, importing MRI images for registration

The iSR'obot Mona Lisa 2.0 is a system with MRI-US fusion function that can provide visualization and locate regions of interest. Using UroFusion software, medical professionals can create personalized biopsy plans and employ a robotic system to guide the biopsy needle with precise target positions, achieving complete prostate coverage. The Eigen/Artemis system supports early detection of prostate cancer through the diagnostic software ProFuseCAD. This system integrates information from various imaging modalities such as mpMRI, positron emission tomography, CT, and contrast-enhanced ultrasound to enhance US fusion, enabling targeted biopsy and treatment. The Invivo Corporation/Uronav (Version 2.0) system integrates pre-biopsy MRI of the prostate with real-time US-guided biopsy images, providing clear visualization of the prostate and suspicious lesions while delineating the biopsy needle path. The Uronav system combines electromagnetic tracking and navigation with onboard computers and real-time imaging interfaces, providing precise positioning for clinical practice in mobile workstations. KOELIS/TRINITY is a fully integrated mobile fusion imaging platform equipped with 3D US probes and KOELIS’ patented organ-based tracking fusion technology, designed specifically for personalized prostate care. Focal Healthcare/Fusion Bx 2.0 enables urologists to complete surgery in a shorter time and with minimal training through a step-by-step workflow. The buttons on the stepper allow urologists to complete most of the operations without having to move their hands away from the probe, and automatic motion compensation adjusts for patient movement to maintain image alignment.

The development of these devices and systems has significantly enhanced the accuracy and efficacy of the theranostic process for prostate cancer, providing medical professionals with enhanced visual guidance and precise positioning capabilities. Natarajan et al. [127] performed FLA surgery on 11 individuals with moderate-risk prostate cancer using MRI-US fusion as guidance. This technology directed laser fibers to the lesion containing prostate cancer via the rectum. Real-time temperature monitoring during LA was achieved using a thermal sensor. Ten of 11 patients were effectively treated with local anesthesia. This study demonstrated the feasibility and safety of using MRI-US fusion for guiding focal LA, highlighting its potential in prostate cancer treatment.

With the development of diagnostic imaging techniques using MRI or MRI-US fusion, the reliable characterization of tumors can provide more accurate diagnostic information, thus enabling more comfortable options like active monitoring and “super active monitoring” or local treatment [128].

LA guided by multimodal optical imaging has achieved significant success [129], especially in applications such as brain tumors. However, endoscopic optical imaging-guided LA remains a research focus. There is a growing need to advance and refine the shared optical path coupling techniques like OCT, PA, and LA to meet the needs of specific clinical applications. Thorough exploration of multimodel medical imaging fusion alongside the strategic design of a proficient robot system serves to resolve the intricate issues surrounding positioning and control within endoscopic laser surgery. These efforts collectively work toward the successful navigation and guidance of surgical procedures, ensuring precision and effectiveness during surgery.

## Robot-Assisted LA

In current clinical practice, LA surgeries are performed using a flexible bronchoscope approach. This technique involves the insertion of an optical fiber through the bronchoscope biopsy. The distal end of the bronchoscope is extended by at least 1 cm, ensuring alignment and maintaining a distance of approximately 4 to 10 mm from the intended ablation target. However, this method lacks automation and real-time monitoring capabilities for the ablation process. At present, there are ongoing clinical applications of the robot-guided platforms for both biopsies and LA interventions. Song et al. [130] designed a new type of biopsy capsule robot driven by an external magnetic field, which can cut and collect intestinal tissue samples without tearing or sticking. Rubino et al. [131] used the MedTech ROSA ONE Brain robot to successfully execute LA procedures for brain tumors in a cohort of 43 patients under MRI guidance, and only one single patient required reoperation, which proved the ROSA robot’s capacity to achieve efficient, safe, accurate, and fast LA treatment.

In order to enhance the stability and precise localization of the surgical ablation area, as well as to enable real-time monitoring of the ablation process, researchers are developing new continuum robotics (CRs) and micro-robot end effectors for advanced LA surgery robot systems. This innovative approach involves positioning the imaging sensors and laser actuators closer to the surgical site, thereby enhancing surgical precision and enabling real-time monitoring of the ablation procedure.

### End effector of micro-robotic LA

The micro-robot is designed to serve as the end effector of an LA system, providing precise laser guidance near the surgical site while offering high-resolution motion and rapid response capabilities. In their work, Lee et al. [132] reviewed robots for LA surgery by controlling optical fibers, and classified them based on their actuation strategies into magnetically driven, motor-driven, and piezoelectric actuated robots. Acemoglu et al. [133] developed a magnetic drive-based laser scanner for high-speed laser scanning in endoscopic minimally invasive procedures. This device employs a magnetic drive to manipulate the laser fiber, enabling precise control over 2D positioning and rapid scanning. This innovation aims to combine the advantages of the free beam laser scanning system with the endoscopic minimally invasive procedures, thereby enhancing the precise and effective treatment in patient. Zhao et al. [134] have developed a novel robot system with a cable-driven parallel mechanism for controlling laser fibers for surgery. This system adopts an innovative mechanical structure, which can independently generate scanning paths or allow user-driven free paths for scanning. Characterized by high precision and repeatability, it offers accurate laser guidance and operation, and gives minimally invasive procedures greater flexibility and control.

**Fig. 10.**
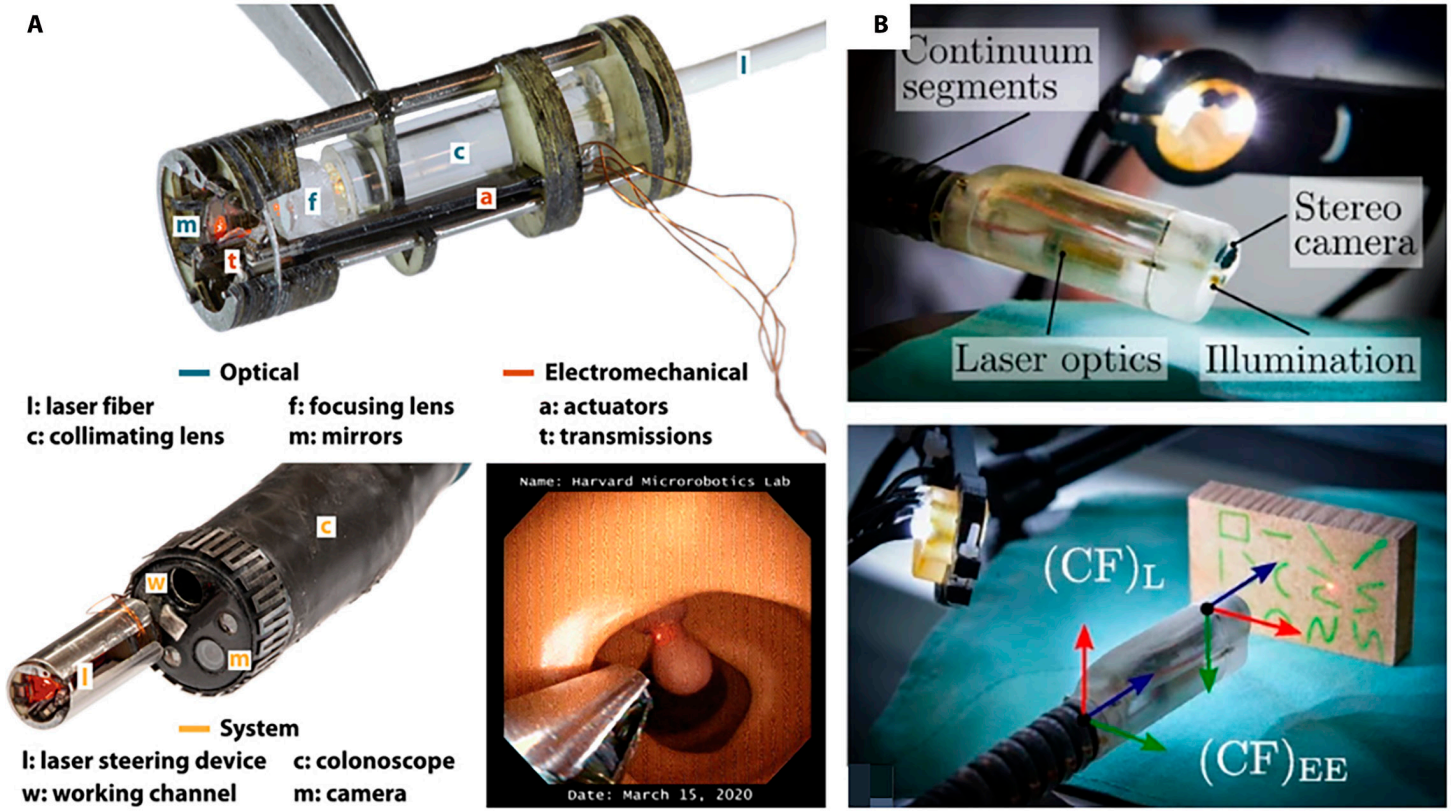
Precision micro-robot end effector. (A) Micro-robot laser steering system. Reproduced with permission from [52]. (B) Endoscopic tip. Reproduced with permission from [53].

Bothner et al. [135] proposed a novel laser scanning system based on a piezoelectric beam-driven micro-electromechanical integrated structure for oral robot surgery. This system employs lasers for tissue cutting during surgery, reducing the collateral damage to healthy tissue. This instrument incorporates the benefits of both laser-based minimally invasive procedure and robot-assisted minimally invasive procedure, and can be operated by a robot arm to achieve more precise cutting and sharper incisions. Subsequently, this device was further miniaturized [52], as illustrated in Fig. 10A, which can control the position and speed of fiber optic laser transmission. It can be affixed to the tips of surgical instruments (such as flexible endoscopes or continuous surgical robots) and can access dissection structures within narrow canal that are typically hard to reach. Moreover, the system is equipped with sensing capabilities and can use laser-based imaging modalities (such as OCT and PA) to visualize deep anatomical structures within the body. This additional information provides surgeons with additional information and visual assistance, enabling them to make more precise decisions. This technology holds the potential to advance the development of minimally invasive procedures and provide patients with improved treatment outcomes and recuperation experiences. Kundrat et al. [53] created an endoscope tips with stereo vision, lighting, and fixed-focus lasers ,as illustrated in Fig. 10B. These specialized tips can be used for noncontact intralaryngolasia surgery and have demonstrated success in pig larynx experiment. The endoscope is anticipated to be further miniaturized in the subsequent stage, allowing for a wider range of clinical applications for the surgical access through the natural openings.

### Continuum robotic LA

**Fig. 11.**
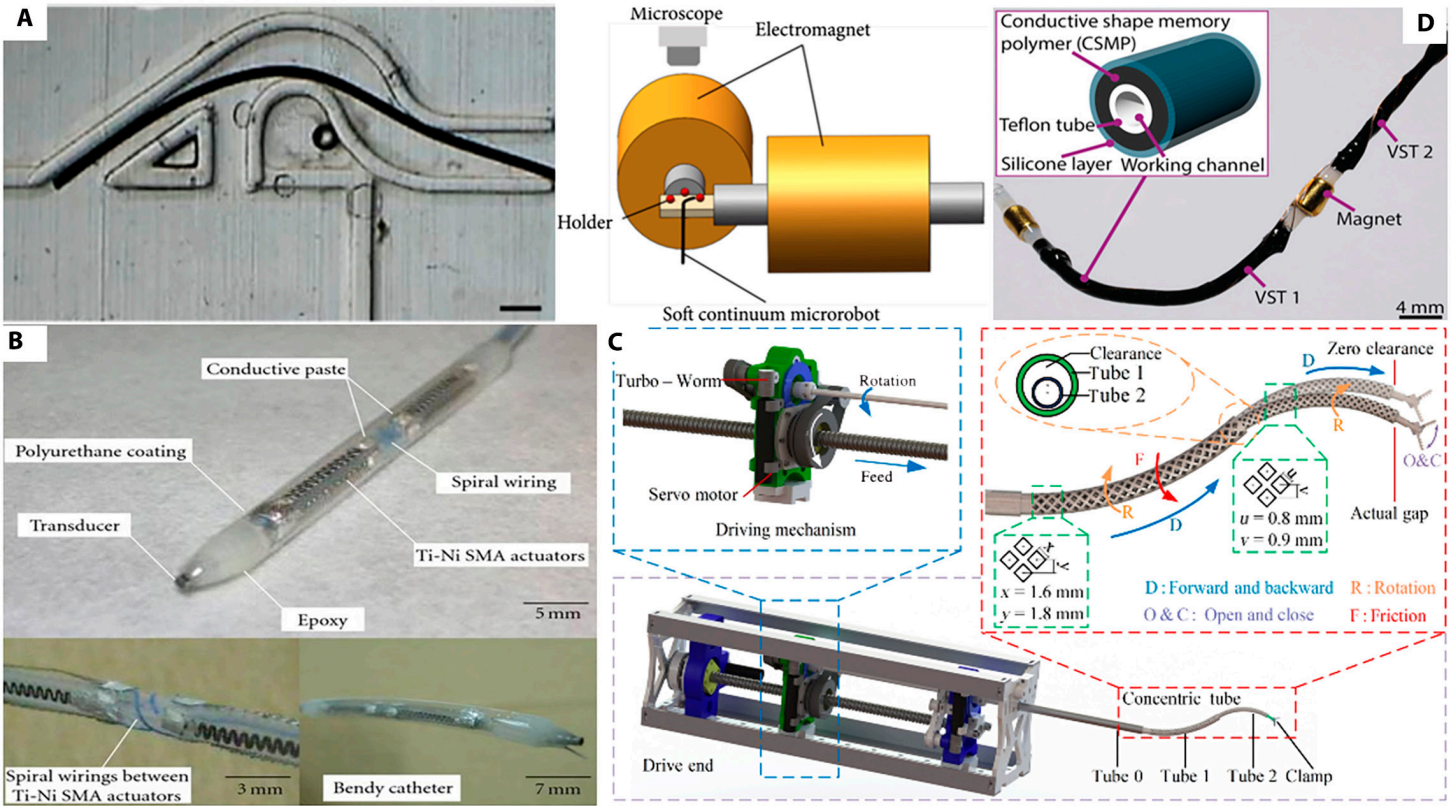
CR for LA of tumor treatment. (A) Magnetic navigation CR. Reproduced with permission from [139]. (B) Shape memory CR. Reproduced with permission from [141]. (C) Concentric tube. Reproduced with permission from [142]. (D) Conductive polymer-driven conduit. Reproduced with permission from [143].

In the LA surgery, the specific purpose of CRs is to provide a robotic framework for deploying, supporting, and correctly positioning imaging and laser steering systems. CRs are robotics whose mechanisms are driven by operators or actuators. Hu et al. [136] reviewed CRs in minimally invasive procedures, outlining their achievements and future applications. According to their driving principles, CRs are divided into four categories: tendon-driven CRs [137,138], magnetic navigation CRs [139,140], soft material-driven CRs (including shape memory effect CRs [141], concentric tubes [142], conducting polymer-driven CRs [143], and hydraulic pressure driven CRs [144]), and hybrid actuation CRs [145], as illustrated in Fig. 11. Chikhaoui et al. [80] have developed a novel biocompatible conductive polymer that can be used in visceral medical robots. This polymer can cause a volume change in the pipeline by applying a reduction or oxidation potential to the component electrode, thereby achieving the desired bending of the pipeline.

However, effective methods for regulating CRs, particularly in terms of end force control, are still a challenge. Although information on tip force can be obtained by designing sensors at the tip, the dependability of CRs may be compromised. In addition, there is a lack of efficient analysis tools for real-time feedback control of CRs, hindering the establishment of dynamic behavior models for CRs. The development of control methods and real-time feedback systems that can manipulate CRs to improve their accuracy and dependability requires additional research and development. This will assist in optimizing CR operation during LA surgery, and provide precise force feedback and control capabilities. Moreover, future research should focus on the creation of new analytical tools and models to better comprehend and predict the dynamic behavior of catheters with enhanced controllability.

### Future directions of robotic-assisted LA

Robot-assisted laser surgery is a challenging and delicate operation that requires the development of corresponding technology development support. This includes tumor identification and diagnosis, exploration of biological effects of laser–tissue interaction, CRs, micro-electromechanical integration for laser steering robotic, tissue tracking, motion control, and real-time intraoperative temperature detection. These are fully integrated into a complete clinical robot system to realize the integration of optical tumor theranostics.

CRs, typically flexible and slender, are usually preferred to accommodate a wide range of patients, with micro-electromechanical integration robots coupled at their ends for coordinated movements for performing precise LA. Equipped with lighting and high-definition imaging systems, the robot integrates advanced 3D vision, thereby optimizing the automatic control and path planning methods for surgical precision. At the end of the CRs, an optical diagnostic probe can also be integrated, coupled with laser beams such as OCT, PA, and LA through the optimization of multimodal algorithms, so as to accurately and quickly identify the edge of tumor tissue and precise LA procedures.

At present, the exploration of combining established technologies with innovative structures is still in its infancy, and a complete robotic system has not yet been realized. Nevertheless, this domain is seen as the focus of future research. Despite the complex changes surrounding the clinical implementation of robotic-assisted LA, its potential benefits are clear to patients, surgeons, hospitals, and the public healthcare system. As these technologies undergo clinical validation, more patients will have the opportunity to undergo LA surgery and benefit from improved surgical precision and quality. Surgeons will benefit from more intuitive surgical arrangements and robotic assistance, which will help them better plan and execute delicate interventions, thereby diminishing the risk of surgical trauma. Furthermore, the application of these technologies will also yield advantages for hospitals, including reducing the surgical complications and recurrence, improving patient satisfaction, and reducing medical costs to a certain extent. However, prior to the clinical integration of these technologies, the aspects of safety, dependability, biocompatibility, and sterilizable characteristics still need to be fully demonstrated. This will be a critical step to ensure its feasibility and successful application.

## Perspectives and Challenges

LA is an emerging discipline in minimally invasive interventional tumor therapy, and its safety and efficacy have been largely confirmed. It stands as a promising surgical alternative for various cancers. Compared to conventional treatment, LA can reduce recovery time, minimize blood loss, and lower risk. However, its limited laser penetration depth restricts its suitability to superficial tumor tissue ablation [146]. Therefore, improving the laser source is one of the future directions of LA, along with expanding the clinical applications of LA to other fields, continuing to investigate the interaction between laser and tissue, elucidating the laser treatment mechanisms of different wavelengths and laser parameters, refining the targeting of tumor laser treatment, and using it to treat new diseases and viral diseases.

This paper focuses on the application of LA to tumors and provides a thorough discussion of the mechanism and biological effects of tumor LA treatment, image guidance, robot assistance, and other aspects. Effective simulation of laser parameters and elucidation of biological effect mechanisms will have a significant impact on the LA effect in terms of the LA mechanism. For precise LA treatment, it is necessary to elucidate the laser tissue interaction mechanism of tumors in various positions and tissue structures. Image guidance can provide essential structural and functional information for preoperative, intraoperative, and postoperative ablation outcomes, thereby providing the technical methods for the accurate and effective evaluation of ablation effects, particularly in preoperative planning, intraoperative navigation, and postoperative evaluation.

Image-guided LA has been proven to have promising prospects in tumor treatment, with confirmed efficacy and safety [74,101,114,127], and is changing the available treatment options for patients. Nevertheless, present clinical cases mostly rely on single-modality imaging for guidance, which may offer limited structural and functional data of afflicted tissues. In addition, image resolution, penetration depth, imaging speed, and field of view (FOV) are important factors for real-time outcome visualization. Consequently, multimodal imaging-guided LA is also one of the future development directions. Multimodal imaging can reduce misdiagnosis rates, enhance the recognition efficiency of tumor tissue, and provide anatomical and functional information of tumors with high spatial/temporal resolution [147]. Integrated multimodal imaging and LA treatment can substantially boost diagnostic and therapeutic efficiency.

As a result of the constant advancement of technology, endoscopically guided surgery is increasingly utilized in clinical tumor resections. However, current feasible image-guided laser surgery systems still encounter numerous obstacles and require extensive research and technological innovation in the years ahead. A prime example is the design of a multimodal endoscopic instrument [148], effectively coupling diagnostic and therapeutic light, providing high-quality optical images for tumor diagnosis and real-time control of laser parameters for precise tissue volume ablation. Moreover, it is also urgent to achieve wide-field imaging for multimodal image fusion and construct an effective macro-micro image fusion for extensive FOV guidance in LA, alongside strategies for tissue tracking and motion compensation via robotic catheters.

As an intelligent optical diagnostic and therapeutic instrument in the future, the implementation of LA in the field of tumor theranostics is becoming increasingly widespread. Robot-assisted LA technology is a research hotspot in the field of cancer treatment at the moment. The autonomous and controllable regulation and precise control of robots can facilitate the efficient execution of invasive fiber optic or integrated theranostics with the advent of artificial intelligence and deep learning. In conjunction with deep reinforcement learning [149–151] and other technical parameters, the LA robot or laser theranostic robot can effectively execute operations in a continuous high-dimensional space, thereby enabling intelligent laser theranostics. Moreover, forming a 3D space of the human body based on multidisciplinary computational anatomy [152] via preoperative and intraoperative image fusion, augmented by surgical navigation-guided autonomous robotic operation strategies [153], will facilitate the efficient operation of laser theranostic robotics. However, more research is required in the field of medical ethics.

Over the past 60 years [154], LA has gradually been widely used in clinical applications. The safety of laser therapy is an aspect that must be considered in tumor treatment [4]. Controlling the laser output dose is an important manifestation of the accuracy of LA. While the journey toward clinical systems might be long, the integration of endoscopic optical theranostics is a development trend. This new therapeutic diagnostic technology can bring significant benefits to medical theranostic applications, particularly in delicate human organ surgeries. This includes the potential for more precise surgical margins in oncology applications as well as safer procedures for better preservation of healthy tissues and essential structures.

## Conclusion

This paper aims to comprehensively review the present status and future directions of tumor LA therapy. The paper first discussed the mechanism and biological effects of LA, followed by an evaluation of image-guided technologies employed in tumor LA therapy. Furthermore, the paper also focuses on LA therapy assisted by surgical robots and puts forward new thinking and perspectives on the prospect of the integration of intelligent multimodal image guidance and LA theranostics.

This paper points out that intelligent multimodal image-guided LA theranostic technology has broad application prospects in the field of tumor LA therapy. By combining a variety of intelligent image-guided technologies, precise guidance and monitoring of the tumor LA process can be achieved, resulting in enhanced treatment accuracy and effect of treatment, and improving clinical efficacy. The expansion of this technology is poised to bolster the extensive application of tumor LA therapy and bring better treatment outcomes for patients.

In general, with the continuous advancement of technology, the fusion of multimodal image guidance with LA theranostics promises revolutionary transformations in the field of tumor therapy. This will not only improve the effect of LA treatment but also empower medical practitioners with more precise treatment plans, which will bring greater benefits to patients' health.

## Data Availability

The data used to support the findings of this study are available from the corresponding author upon request.
